# Structure-effect relationship of phenolic compounds on *α*-amylase inhibition studied by isothermal titration calorimetry

**DOI:** 10.1016/j.crfs.2025.101266

**Published:** 2025-12-06

**Authors:** Mengyao Xiong, Jörn Plambeck, Tuba Esatbeyoglu, Maria Buchweitz

**Affiliations:** a Department of Chemistry, Institute of Food Chemistry, University of Hamburg, Hamburg, 20146, Germany; b Department of Molecular Food Chemistry and Food Development, Institute of Food and One Health, Leibniz University of Hannover, Hannover, 30167, Germany

**Keywords:** Inhibition mechanism, Starch hydrolysis, Digestive enzymes, Aronia, Apples, Tea, Black carrot, Polyphenols

## Abstract

Recent studies have emphasized the therapeutic potential of polyphenols in regulating blood glucose levels. To assess the inhibitory effects of different phenolic structures and various plant extracts on pancreatic *α*-amylase in real-time under physiologically relevant conditions, starch hydrolysis was monitored by isothermal titration calorimetry. Significant impact of the phenolic structure was observed for the inhibition strength and mechanism. While some polyphenols demonstrated reversible inhibition, others seem to form aggregates. Traditional apple varieties exhibited greater *α*-amylase inhibition than commercial varieties. Apple peel, characterized by higher phenolic contents and flavonols, exceeded the inhibition by extracts from flesh. Aronia rich in procyanidins and tea with EGCG revealed excellent and concentration-dependent inhibition. In contrast to cyanidin-3-*O*-glucoside, black carrot anthocyanins exhibited insignificant inhibition, indicating a limiting impact of the sugar acylation. These findings underscore the role of specific polyphenol structures in modulating carbohydrate digestion, suggesting their potential for managing glycaemic control.

## Highlights

• Continuous monitoring of the time dependent conversion of the **natural** substrate starch and thus avoiding artefacts due to the use of artificial saccharides (e.g. modified with a chromophore usually)
• Inhibition of amylase under conditions relevant for digestion (buffer, pH, concentrations)
• Relationship between the structure and the inhibitory effect of purified standard compounds
• Inhibitory effects of complex coloured/turbid plant extracts (tea, aronia and apple) and fractions thereof combined with qualitative and quantitative analyses of individual phenolics
• Translating academic knowledge into clear nutritional recommendations.

## Graphical abstract

**Figure:**
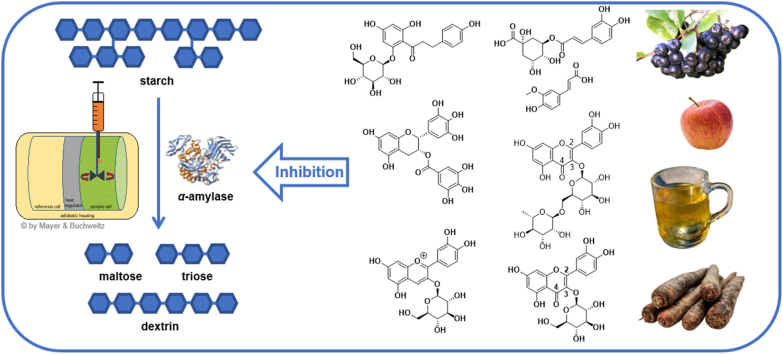

## Abbreviations

CAT: (+)-catechin
EC: (−)-epicatechin
PC B1: procyanidin B1
PC B2: procyanidin B2
PC C1: procyanidin C1
FA: ferulic acid
PHL: phlorizin
CYD-3-glc: cyanidin-3-O-glucoside
Q-3-glc: quercetin-3-O-glucoside
rutin: quercetin-3-O-rutinoside
EGCG: epigallocatechin gallate
SD: standard deviation
ITC: isothermal titration calorimetry
EI: enzyme inhibitor complex
ESI: enzyme substrate inhibitor complex
*K*_*d*_: dissociation constant
*K*_*ic*_: competitive inhibition constant
**K**_**iu**_: uncompetitive inhibition constant
**K**_**m**_: Michaelis-Menten constant
**IC**_**50**_: half maximal inhibitory concentration
*v*_*max*_: maximal reaction rate
MW: molecular weight
*Q*: heat
*S*: substrate
${\Delta_{R}H}_{app}$: experimentally determined molar enthalpy
*dQ/dt*: heat rate
GalG_2_CNP: chloronitrophenyl-galactopyranosylmaltoside
CNP: chloronitrophenol
PAHBAH: 4-hydroxybenzoic acid hydrazide
DNS: 3,5-dinitrosalicylic acid
***p***: NP *p*-nitrophenol
G_2_-*p*NP: *p*-nitrophenyl-α-D-maltoside
SPE: solid phase extraction
DW: dry weight

## 1 Introduction

Diabetes mellitus type 2 is a chronic metabolic disorder characterized by insulin resistance and impaired glucose metabolism. Its development is multifactorial, with a significant impact of a carbohydrate-rich diet, which can lead to spikes in blood glucose levels after meals, known as postprandial hyperglycemia (Ayua et al., 2021; Ryu et al., 2014; Rolo and Palmeira, 2006). In recent years, a polyphenol-rich diet has been promoted as a potential approach for the enhancement of insulin sensitivity (Williamson and Sheedy, 2020; Shahwan et al., 2022) and the regulation of blood glucose levels (Shahwan et al., 2022; Moser et al., 2018). Research has indicated that polyphenols inhibit key digestive enzymes, such as *α*-amylase and *α*-glucosidase, which play critical roles in starch hydrolysis. While *α*-amylase is essential for initiating the breakdown of starch, *α*-glucosidase facilitates the final step of the glucose formation. Inhibiting both enzymes and thereby slowing the processes might help to reduce glucose spikes in the bloodstream after meals, which is important since elevated blood glucose levels increase the risks of serious complications, including damage to the eyes, the kidneys, the nerves, the heart, and the peripheral vascular system (Yilmazer-Musa et al., 2012; Kim et al., 2000; Adisakwattana et al., 2009).

**Fig. 1:**
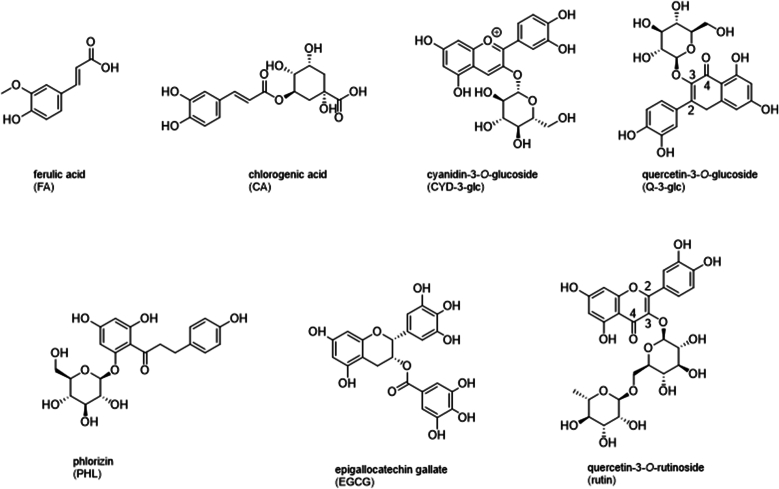
Chemical structures of polyphenols investigated in the study.

It is well known that specific structural features of phenolics have a significant impact on *α*-amylase inhibition. Xiao et al. summarized the following key points (Xiao et al., 2013): (i) Methylation, (ii) methoxylation and (iii) glycosylation of hydroxyl groups in flavonoids weaken the inhibitory effects. Conversely, (iv) hydroxylation, (v) galloylation, and (vi) the presence of an unsaturated 2,3-bond coupled with a 4-carbonyl group in flavonoids (Fig. 1) enhance the inhibition. Furthermore, Kaeswurm et al. found out that the molecular weight and the extension of the aromatic system significantly influence *α*-amylase inhibition (Claasen et al., 2025; Kaeswurm et al., 2019). This explains why epigallocatechin gallate (EGCG) (Kaeswurm et al., 2019; Jia et al., 2019), a flavonoid containing a galloyl group, and oligomeric procyanidins (Claasen et al., 2025), which consist of flavan-3-ol units up to 10 (Gu et al., 2011), are the most effective inhibitors among all flavonoids (Kaeswurm et al., 2019; Narita and Inouye, 2011; Sui et al., 2016; Nyambe-Silavwe and Williamson, 2018; Nyambe-Silavwe et al., 2015; Akkarachiyasit et al., 2010; Le et al., 2024). Moreover, the inhibitory effects of plant extracts have been extensively reported (Berger et al., 2020; Sun et al., 2016a, 2016b, 2019; Kaeswurm et al., 2020). However, neither did they compare the inhibitory effects to specific phenolic fractions nor to individual phenolic structures. Furthermore, often unrealistic concentrations or conditions might confound the study outcome, and it is noteworthy that most studies rely on artificial substrates instead of natural starch, which have an impact on substrate binding and thus on enzyme activity. Therefore, due to their high phenolic content, apples, predominantly containing monomeric and oligomeric flavan-3-ols (Stanger et al., 2017), and aronia berries, which are rich in anthocyanins, chlorogenic acids and procyanidins (Kennedy and Jones, 2001; Rodríguez-Werner et al., 2019), have been selected to investigate their ability to modulate enzyme activity. Furthermore, green and black tea, known for its abundance of catechins (Sun et al., 2016a), and black carrot containing high levels of acylated anthocyanins (Montilla et al., 2011) have been studied.

In our previous study, an innovative approach to evaluate the inhibitory effects of flavan-3-ols on *α*-amylase using isothermal titration calorimetry (ITC) has been developed (Claasen et al., 2025). This approach enables continuous monitoring of starch hydrolysis. Traditional enzyme inhibition assays, which are predicated on the use of artificial substrates and frequently rely on UV/Vis spectroscopy for product detection, give rise to certain issues (Todd et al., 2001). The use of complex and colloidal extracts in such assays can pose significant challenges and lead to several difficulties. The turbidity of extracts might cause light scattering, resulting in potential distortion of the absorption readings. Furthermore, if an extract exhibits strong absorption in a specific wavelength range, it may interfere with or mask the detection of other compounds. In contrast, ITC has low demands on substrate and reaction conditions and is therefore suitable for a wide range of nutritionally relevant substrates and a complex environment. Moreover, ITC allows for continuous monitoring of starch hydrolysis, capturing the dynamic nature of enzyme activity.

The aim of the present study is to evaluate the inhibitory effects of different phenolic structures (Fig. 1) and complex plant extracts on *α*-amylase activity to understand their potential as natural therapeutic agents assisting in the management of blood glucose levels. The ITC approach enables the determination of key parameters such as reduction in enzyme activity *(v*_max_ [%]), change in reaction enthalpy $({\Delta_{R}H}_{app}$[%]), inhibition constants (*K*_*ic*_, *K*_*iu*_), and half maximal inhibitory concentration (*IC*_*50*_), providing detailed kinetic data to deepen our understanding of enzyme activity and inhibition mechanisms.

## 2 Experimental section

### 2.1 Chemicals

Disodium hydrogen phosphate (anhydrous, ≥99 %) and sodium dihydrogen phosphate dihydrate (≥99 %) were sourced from Carl Roth (Karlsruhe, Germany). Sodium chloride (99.5 %) was purchased from Grüssing (Filsum, Germany). *α*-Amylase (from porcine pancreas, 22 mg/mL, 1180 units/mg protein, lot SLCJ3459) and starch from potato (lot 0000221448) were obtained from Sigma Aldrich (Taufkirchen, Germany). Chlorogenic acid (CA, 96 %, lot WXBD5681V), phloridzin dihydrate (PHL, 100 %, lot SLBP3626V), epigallocatechin gallate (EGCG, 96 %, lot SLCK1721), ferulic acid (FA, 99.8 %, lot STBC5005V) and (−)-epicatechin (EC, 97 %, lot BCCD3979) were also sourced from Sigma Aldrich. (−)-Epicatechin-3-gallate (99.4 %, lot 26963), quercetin-3-*O*-glucoside (Q-3-glc, 92 %, lot 12267), cyanidin-3-*O*-glucoside chloride (CYD-3-glc, 99.7 %, lot 13053), cyanidin-3-*O*-galactoside chloride (99.3 %, lot 14876) were obtained from Phytolab (Vestenbergsreuth, Germany). Additionally, quercetin-3-*O*-rutinoside (rutin, ≥97 %, lot A0299493) were purchased from Acros Organics (New Jersey, United States). Caffeine solution standards (500 μg/mL) in a 1:1 mixture of acetonitrile and methanol were obtained from Ultra Scientific (North Kingstown, United States). Acetonitrile (UHPLC supergradient, >99.9 %) was acquired from PanReac AppliChem ITW Reagents (Darmstadt, Germany). Formic acid (≥99 %) for HPLC analysis was obtained from VWR Chemicals (Darmstadt, Germany) and acetic acid (100 %) was ordered from Carl Roth (Karlsruhe, Germany).

### 2.2 Polyphenol preparation

All polyphenol standards were dissolved in 0.1 M phosphate buffer (pH 7.0, containing 0.04 M NaCl) and then diluted 1:100 with 0.1 M phosphate buffer (pH 7.5). Concentrations were determined using UV/Vis spectroscopy at 298 K (SPECTROstar Nano, BMG Labtech, Germany) with a quartz glass cuvette (d = 1 cm, Hellma Analytics, Germany). The following extinction coefficients (*ε*), based on Kaeswurm et al. (2021) were used: *ε*_280nm_ (EC) = 3470 [L/mol∗cm], *ε*_280nm_ (CA) = 9200 [L/mol∗cm], *ε*_280nm_ (EGCG) = 10,400 [L/mol∗cm], *ε*_280nm_ (PHL) = 8100 [L/mol∗cm], *ε*_280nm_ (FA) = 13,600 [L/mol∗cm], *ε*_280nm_ (Q-3-glc) = 14,300 [L/mol∗cm] and *ε*_280nm_ (rutin) = 14,300 [L/mol∗cm] (same as Q-3-glc, due to the same aglycone structure and negligible impact of the sugar in absorption), *ε*_510nm_ (CYD-3-glc) = 27,000 [L/mol∗cm]. All the solutions were stored at 253 K. Oxidation of epicatechin (EC_ox_) was performed by stirring the EC stock solution (c = 4 mM, V = 2 mL) on air at room temperature for 24 h. Following this period, 0.1 M phosphate buffer (pH 7.0, containing 0.04 M NaCl) was added to restore the initial volume.

### 2.3 Apple polyphenols

The study included peel and flesh tissues from five different apple varieties harvested in 2019, including two traditional varieties (Bohnapfel and Gewürzluiken) and three commercially relevant varieties (Granny Smith, Golden Delicious, and Santana). Phenolic extracts from flesh (1.5 g in 2 mL of 0.01 % aqueous HCl) and peel (0.5 g in 2 mL of 0.01 % aqueous HCl) were prepared according to Kaeswurm et al. and stored at −20 °C until use (Kaeswurm et al., 2022, 2023). Information about the individual phenolic structures and contents of the investigated varieties are available in the Supporting Information (S1-2). For the α-amylase activity assays, the flesh extracts were diluted to a ratio of 1:1,875, while the peel extracts were diluted at 1:625, resulting in a final concentration of 0.4 g/L for both, using 0.1 M phosphate buffer (pH 7.0, containing 0.04 M NaCl).

### 2.4 Aronia polyphenols

Aronia extracts were obtained from *Aronia melanocarpa* (black chokeberry) juice through adsorption chromatography using an Amberlite XAD-7 column according to Kostka et al. (2020). To separate the anthocyanins from other phenolic compounds, fractions were generated using a Sartobind S IEX 150 mL cellulose membrane from Sartorius (Göttingen, Germany). The solvents were then removed in vacuo and the residues were freeze-dried. The HPLC-PDA chromatograms and quantification data for the complex XAD-7 extract, the colorless phenolic fraction, and the anthocyanin fraction, are presented in S3-6. *Aronia melanocarpa* contains mainly cyanidin-3-O-galactoside and cyanidin-3-O-arabinoside, along with minor amounts of cyanidin-3-O-glucoside and cyanidin-3-O-xyloside. Among the colorless phenolic compounds, neochlorogenic acid and chlorogenic acid are identified as the major constituents. For the *α*-amylase activity assay, aronia extracts were prepared at varying concentrations of 0.008 g/L, 0.016 g/L and 0.032 g/L, each dissolved in 0.1 M phosphate buffer (pH 7.0) containing 0.04 M NaCl.

### 2.5 Tea polyphenols

Green tea was purchased from J.J. DARBOVEN (Hamburg, Germany) and black tea was obtained from Formosa Phoenix Tea Farm Co., Ltd. (Chaozhou, China). Two grams of tea leaves (black and green) were extracted with 200 mL of deionized water. The extraction process was performed at 100 °C for 3 min for black tea and at 80 °C for the same duration for green tea. In addition, to investigate the impact of steeping time on inhibitory effects, 2 g of black tea leaves were extracted with 200 mL of deionized water at 100 °C for 10 min. All tea extracts were sealed and stored at −20 °C until needed for further use. The HPLC-PDA chromatograms and quantification data for the tea extracts are provided in S7-8. Green tea contains a higher polyphenol content than black tea, with EGCG identified as the primary component, followed by epicatechin-3-gallate and EC. Black tea contains primarily EGCG and epicatechin-3-gallate. However, no EC was found in black tea. Furthermore, black tea exhibits a higher polyphenol content in extracts steeped for longer durations. For ITC experiments, the tea extracts were further diluted with 0.1 M phosphate buffer containing 0.04 M NaCl to reach a final concentration of 0.13 g/L and 0.25 g/L.

### 2.6 Black carrot polyphenols

Solid phase extraction (SPE) of the black carrot concentrate (1247 g/L; estimated by weighing the liquid extract) donated by Wild (Valencia, Spain) was performed following the protocol established by Kaeswurm et al. to remove sugars, organic acids, and colorless phenolics from crude phenolic extracts (Kaeswurm et al., 2020). After SPE purification, the anthocyanin eluate was concentrated using a rotary evaporator, re-diluted with acidified water (0.01 % HCl), and stored at −20 °C until needed. The HPLC-PDA chromatograms and quantification data for black carrot concentrate and anthocyanin extract are available in the Supporting Information (S9-10). For the ITC experiments, the black carrot concentrate and anthocyanin extract were further diluted 1:12 in 0.1 M phosphate buffer containing 0.04 M NaCl to achieve a final concentration of 98 g/L, which corresponds to a molar concentration of 100 μM, matching the concentration used for CYD-3-glc.

### 2.7 α-Amylase preparation

The α-amylase stock solution was prepared according to Classen et al. (Claasen et al., 2025). An α-amylase suspension (20 mg/mL, 1180 units/mg) was diluted 1:13,333 with 0.1 M phosphate buffer (pH 7.0) containing 0.04 M NaCl to achieve a final concentration of 26 nM for the experiments. The amylase stock solution was diluted each day and stored during the day at 4 °C.

### 2.8 Optimizing injection volume in ITC assays

The experiments were conducted using an Affinity ITC LV instrument with a cell volume of 182 μL (TA Instruments, Eschborn, Germany). A starch solution was prepared daily following the protocol recommended by Claasen et al. (2025). This solution was injected into 26 nM α-amylase in 0.1 M phosphate buffer/0.04 M NaCl at 310 K. Injection volumes ranged from 4 to 7 μL, with a single injection per volume and an interval of 1600 s between injections. These controls were conducted to determine the optimal injection volume to achieve maximal velocity (*v*_*max*_) (S11). To minimize interference from product inhibition (Claasen et al., 2025), 5 μL were injected to evaluate the inhibitory effects of polyphenol standards and extracts on α-amylase. Control experiments without inhibitor were performed at the beginning and end of each day to confirm the stability of α-amylase during the day. To investigate the inhibitory effects, 26 nM α-amylase was pre-incubated with 100 μM polyphenols (except for EGCG, which was used at 25 μM) and polyphenol extracts (apple extracts at 0.4 g/L; aronia extracts at 0.008 g/L, 0.016 g/L and 0.032 g/L; tea extracts at 0.13 g/L and 0.25 g/L; black carrot extracts at 98 g/L) for 1 h at 277 K before adding starch. Enzyme kinetic constants were determined at 310 K.

The heat released during starch conversion was continuously monitored, with calculations performed using Microsoft Excel® software. The enzymatic rate (*v*) is directly proportional to the measured thermal power (eq (1)).

(1) $$v=\frac{dP}{dt}=\frac{1}{V_{cell}\;\cdot \;{\Delta_{R}H}_{app}}\;\cdot \;\frac{dQ}{dt}$$

The reaction enthalpy (${\Delta_{R}H}_{app}$) is determined by integrating the heat peak over its entire duration, divided by the number of substrate molecules converted:

(2) $${\Delta_{R}H}_{app}=\frac{\int_{t=0}^{\infty }\frac{dQ}{dt}\;dt}{n_{S}}$$

*(dQ/dt)*_*max*_ [%], ${\Delta_{R}H}_{app}$ [%] und *v*_max_ [%] are calculated in relation to daily control.

The substrate concentration at time t ([S]_t_) is calculated according to eq (3).

(3) $${\left[S\right]}_{t}=\frac{\int_{t}^{\infty }\frac{dQ}{dt}\;dt}{\int_{0}^{\infty }\frac{dQ}{dt}dt}\;\cdot \;{\left[S\right]}_{t=0}$$

The Michaelis-Menten constant (*K*_*m*_) is the substrate concentration at the time t_0.5_ when the reaction rate is half of the maximum velocity ($v_{\max}$) (see eq (4)).

(4) $$K_{m}=\frac{\int_{t0.5}^{\infty }\frac{dQ}{dt}\;dt}{\int_{0}^{\infty }\frac{dQ}{dt}dt}\;\cdot \;{\left[S\right]}_{t=0}$$

Therefore, the thermogram contains all the required data for calculating inhibition constants. The maximal conversion rate $v_{\max}$ and the Michaelis-Menten constant $K_{m}$ are determined from control experiments conducted in the absence of an inhibitor. In the presence of an inhibitor [*I*] the apparent values of $v_{\max}^{app}$ and $K_{m}^{app}$ are obtained. Competitive inhibition (*K*_*ic*_, eq (5)) is due to the formation of an enzyme-inhibitor complex (EI), whereas uncompetitive inhibition (*K*_*iu*_, eq (5)) occurs when an inhibitor binds to a ternary enzyme-substrate-inhibitor complex (ESI):

(5) $$K_{ic}=\frac{\frac{v_{\max}^{app}}{K_{m}^{app}}\;\cdot \;\left[I\right]}{\left(\frac{v_{\max}}{K_{m}}\right)-\left(\frac{v_{\max}^{app}\;}{K_{m}^{app}\;}\right)}\;K_{iu}=\frac{v_{\max}^{app}\;\cdot \;\left[I\right]}{v_{\max}-v_{\max}^{app}}$$

The *IC*_*50*_ value, a key pharmacological metric, is used to compare the potency of inhibitors. It represents the inhibitor concentration required to reduce enzyme activity by 50 %, which approaches the *K*_*iu*_ value at high substrate concentrations (eq (6)).

(6) $${IC}_{50}=\frac{\left(K_{m}+\left[S\right]\right)}{\left(\frac{K_{m}}{K_{ic}}\right)+\left(\frac{\left[S\right]}{K_{iu}}\right)\;}$$

In order to present the results in a widely accepted format, the thermograms were converted to a Michaelis-Menten plot. The conversion rate (*v*_*0*_) was calculated from the thermogram according to eq (1), and subsequently plotted against the increasing substrate concentration ([*S*])

### 2.9 Statistical analysis

All inhibition assays were conducted at least in duplicate. Data processing was performed using Microsoft Excel 2021 for Windows. Results are expressed as means ± standard deviation (SD). Statistical significance and group differences were analyzed using one-way analysis of variance (ANOVA), followed by Tukey-Kramer Post Hoc Test. Differences were considered significant at *p* < 0.05.

## 3 Results

### 3.1 Inhibitory effects of phenolic structures on *α*-amylase activity

**Fig. 2:**
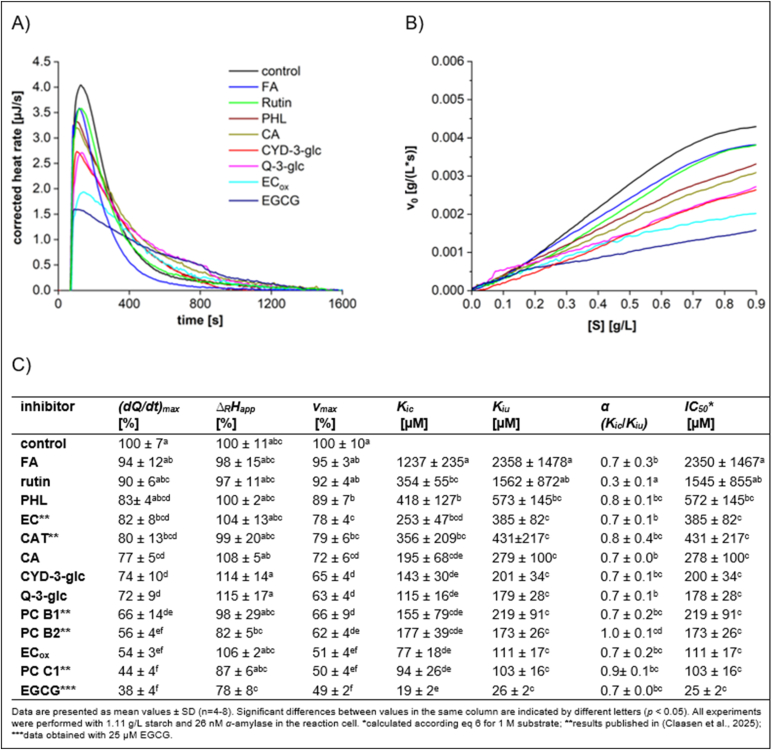
Characteristics of starch conversion by *α*-amylase incubated with 100 μM polyphenols, determined by ITC. (A) Thermogram, (B) Michaelis-Menten diagram, and (C) calculated parameters.

The inhibitory effects on porcine pancreatic *α*-amylase were evaluated by comparing the maximum heat rate and reaction enthalpy shifts in the presence of phenolic compounds with a variety of structural features (e.g., FA for hydroxycinnamic acids, rutin and Q-3-glc for flavonols, PHL for chalcones, CA for hydroxybenzoic acids, CYD-3-glc for anthocyanins, and EC_ox_ and EGCG for flavan-3-ols) to a control without inhibitor. Inhibition strength was evaluated based on *v*_max_, *(dQ/dt)*_*max*_ and *IC*_*50*_ values. In addition, ${\Delta_{R}H}_{app}$, *K*_*ic*_, *K*_*iu*_, and the *K*_*ic*_*/K*_*iu*_ ratios were calculated to provide insights into the underlying inhibition mechanisms (Fig. 2).

The inhibitory strength is most effectively demonstrated by the reduction in both *v*_max_ and *(dQ/dt)*_*max*_ relative to the control. Reversible inhibition is characterized by a constant change in reaction enthalpy ${\Delta_{R}H}_{app}$. As the heat released during the reaction is directly proportional to the amount of product formed, this constant change in ${\Delta_{R}H}_{app}$ indicates that starch can still be completely hydrolyzed into products, even at reduced hydrolysis rates. In contrast, a decrease in ${\Delta_{R}H}_{app}$ indicates that less starch is converted, which is due to the irreversible formation of α-amylase-polyphenol aggregates (Claasen et al., 2025). In this context, since *v*_max_ is significantly influenced by ${\Delta_{R}H}_{app}$ (eq (1)), *(dQ/dt)*_*max*_ is a more reliable representation of the inhibition. The inhibitory strength according to *α*-amylase activity can be summarized as follows: EGCG > PC C1 > EC_ox_ > PC B2 > PC B1 > Q-3-glc ≈ CYD-3-glc > CA > CAT > EC > PHL > rutin > FA. The slight decline in *Δ*_*R*_*H*_*app*_ observed in the presence of PC B2, PC C1 and EGCG suggested a reduction in substrate conversion, indicating the formation of irreversible aggregation between *α*-amylase and polyphenol. For all other polyphenols, *Δ*_*R*_*H*_*app*_ remained nearly constant, with only minor fluctuations likely due to inconsistencies in sample preparation, indicating that the inhibitions were reversible.

This trend in inhibition for *v*_max_ and *(dQ/dt)*_*max*_ was further supported by the calculated inhibitory constants. A lower value of *K*_*ic*_ or *K*_*iu*_ reflects stronger inhibition. Additionally, the *K*_*ic*_*/K*_*iu*_ ratio, represented as the *α* value, provides insight into the mechanism of inhibition. An *α* value of 1 indicates a pure mixed inhibition, and a value lower than 1 a predominant competitive proportion, where the polyphenols preferentially interact with the enzyme rather than binding to the enzyme-substrate complex. Therefore, as the substrate concentration increases, binding of the substrate hinders the additional binding of the polyphenol and at a substrate concentration of 1 M, the *IC*_*50*_ value is equivalent to the *K*_*iu*_ value. EGCG exhibited the lowest values for *K*_*ic*_, *K*_*iu*_, and *IC*_*50*_ (<30 μM), confirming its strong inhibition. In contrast, FA and rutin demonstrated the weakest inhibitory properties, with *IC*_*50*_ values exceeding 1000 μM. Furthermore, the more pronounced fluctuations (SD) due to lower binding affinity are characteristic for weak inhibitors. The *K*_*ic*_*/K*_*iu*_ ratios suggested that rutin, EC, CA, Q-3-glc, CYD-3-glc and EGCG preferentially bind to the enzyme (*K*_*ic*_ < *K*_*iu*_), while the other polyphenols revealed pure mixed inhibition with similar *K*_*ic*_ and *K*_*iu*_ values (α ∼ 1). The inhibitor strength (*IC*_*50*_) was plotted against the molecular weight (S12). No correlation between the molecular weight of polyphenolic compounds, their aglycone and the conjugated system and the inhibitory effects were observed (R^2^ 0.0448–0.1628). Unexpectedly, the data obtained in this study did not align with those reported by Kaeswurm et al. (2019), who found that the *IC*_*50*_ values correlate with the molecular weight of the conjugated system. This discrepancy may be due to the broader range of polyphenols examined in our study, as well as differences in the substrates and the methods employed for investigation.

The rate for starch conversion increased gradually with increasing substrate concentration (Michaelis-Menten diagram), reaching a maximum rate (**v**_*max*_ at *[S]*_*_t=0_*_) in the absence of an inhibitor. The amount of starch added to the enzyme in the ITC experiment was optimized to obtain maximum velocity of substrate conversion while minimizing product inhibition. However, in the presence of an inhibitor, the curve increased more slowly, resulting in a reduced conversion rate than for the control. This observation is consistent with the expected effect of mixed inhibition, where the inhibitor decreases enzymatic activity by binding to both the enzyme and the enzyme-substrate complex.

### 3.2 Inhibitory effects of apple extracts on *α*-amylase activity

#### 3.2.1 Impact of different extract concentration

**Fig. 3:**
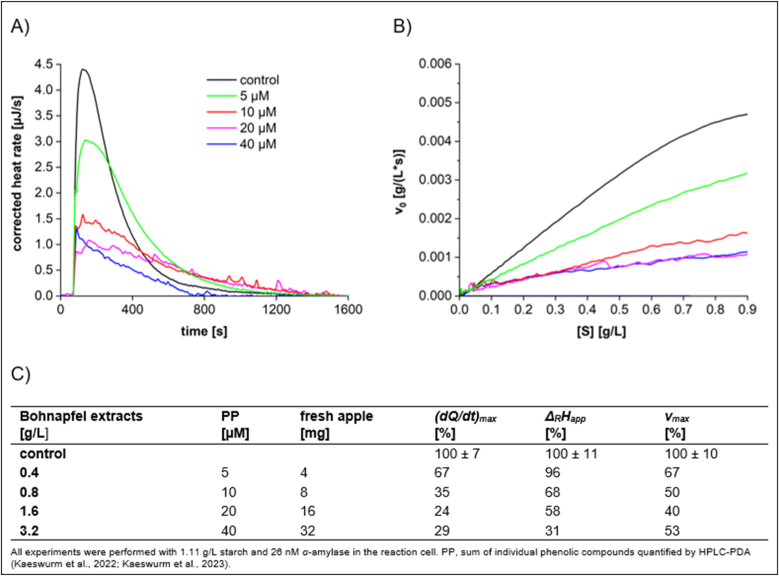
Characteristics of starch conversion by *α*-amylase incubated with different dilutions of **Bohnapfel flesh extract**, determined by ITC. (A) Thermogram, (B) Michaelis-Menten diagram, and (C) calculated parameters.

To evaluate the effect of varying polyphenol concentrations in apple extracts on their inhibition, the thermograms, Michaelis-Menten diagrams, and derived kinetic parameters for the flesh of Bohnapfel at different concentrations are presented in Fig. 3.

With increasing polyphenol concentration, a more pronounced inhibitory effect was observed, as indicated by a decrease in *(dQ/dt)*_*max*_. At a polyphenol concentration of 5 μM, equivalent to a fresh apple mass of 4 mg, the enthalpy change (*Δ*_*R*_*H*_*app*_) remained similar to the control. However, a significant decline in *Δ*_*R*_*H*_*app*_ with increased phenolic contents was noted, indicating a high level of irreversible aggregation between polyphenols and *α*-amylase. Furthermore, the thermograms are smooth and consistent at lower polyphenol concentrations, whereas at higher concentrations a zigzag pattern was observed. This might be attributed to the formation of polyphenol–*α*-amylase aggregates, which interferes with the reaction heat resulting in irregular calorimetric signals (Smith et al., 2022). The correlations of *(dQ/dt)*_*max*_ and *Δ*_*R*_*H*_*app*_ with polyphenol concentration are provided in S13. In contrast to *(dQ/dt)*_*max*_ (R^2^ = 0.4179), a linear correlation (R^2^ = 0.8522) was observed for *Δ*_*R*_*H*_*app*_.

#### 3.2.2 Impact of the apple variety

**Fig. 4:**
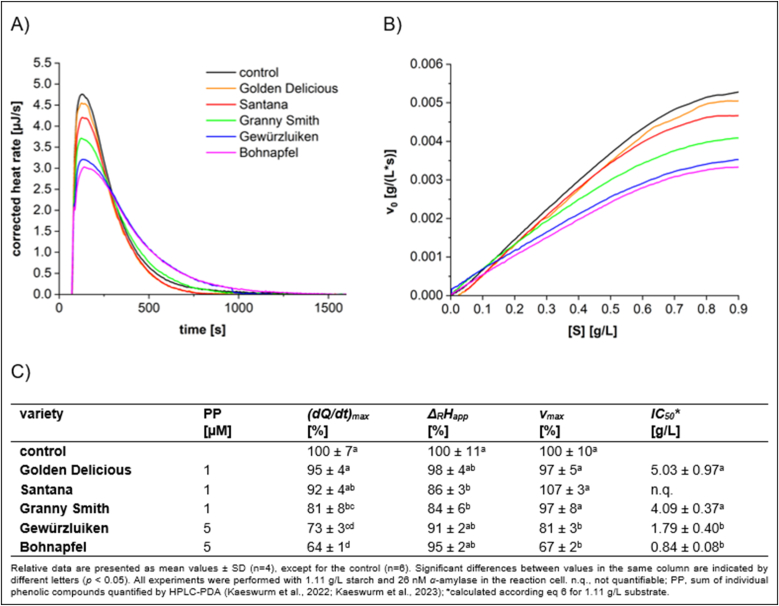
Characteristics of starch conversion by *α*-amylase incubated with **apple flesh** extracts, determined by ITC. (A) Thermogram, (B) Michaelis-Menten diagram, and (C) calculated parameters.

**Fig. 5:**
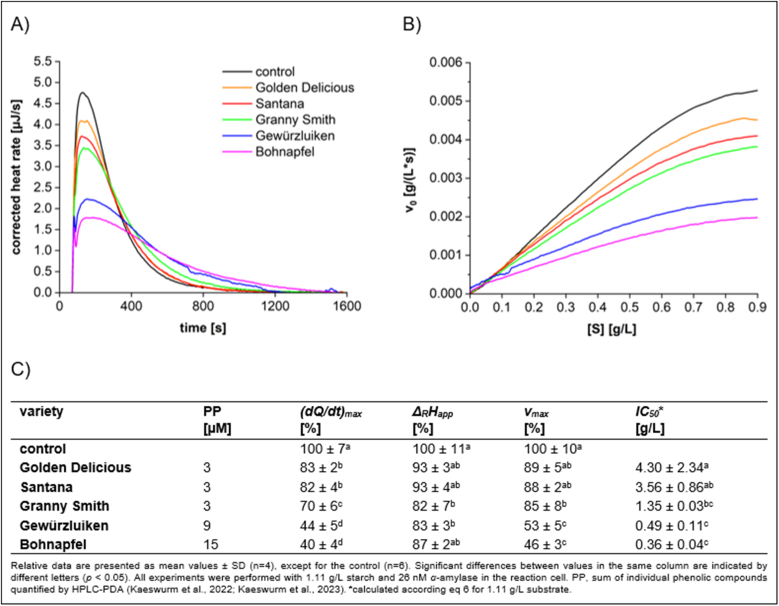
Characteristics of starch conversion by *α*-amylase incubated with **apple peel** extracts, determined by ITC. (A) Thermogram, (B) Michaelis-Menten diagram, and (C) calculated parameters.

Traditional apple varieties are generally rich in polyphenols, which contribute to their astringency and bitterness. In contrast, commercial apple varieties have been selectively bred to reduce polyphenol content, as these compounds are associated with undesirable sensory characteristics (Kaeswurm et al., 2023). To compare the inhibitory effects of different apple varieties, the extract mass concentration was set at 0.4 g/L, ensuring the inhibition was within the reversible range (Fig. 4, Fig. 5).

A strong inhibition of *α*-amylase was observed with polyphenol-rich extracts from traditional apple varieties (S1-2), particularly with Bohnapfel exhibiting the most pronounced inhibition. In contrast, extracts from commercial apple varieties, such as Granny Smith, Golden Delicious, and Santana, which are characterized by lower total phenolic contents displayed significantly reduced inhibition. Moreover, an improved inhibition was observed by apple peels relative to the flesh, which is attributed to the higher phenolic content in the peel as well as variations in the phenolic composition between the peel and the flesh.

To further investigate the relationship between total phenolic content and inhibitory activity, the correlation between *(dQ/dt)*_*max*_ and phenolic content [μg/L] in extracts of both apple flesh and peel was analyzed (S14). A linear correlation was observed for both apple flesh (R^2^ = 0.8952) and apple peel (R^2^ = 0.8521), indicating that higher polyphenol levels are associated with stronger inhibitory effects.

#### 3.2.3 Impact of the individual phenolic profile

**Fig. 6:**
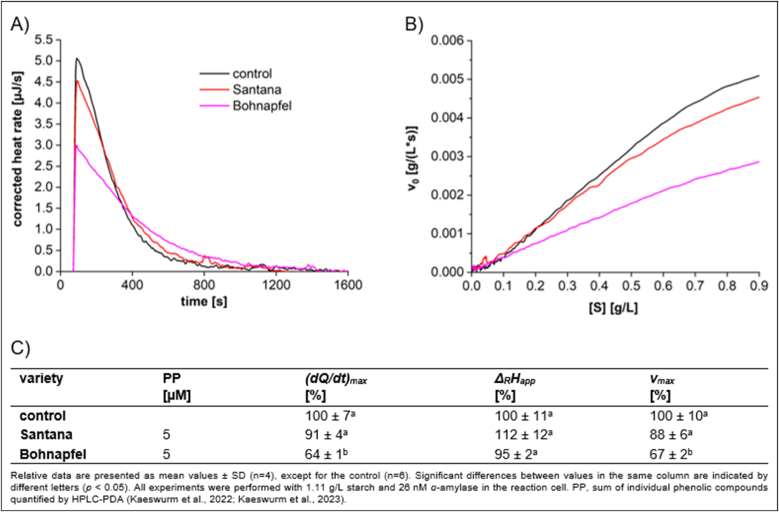
Characteristics of starch conversion by *α*-amylase incubated with equal polyphenol concentration of **Bohnapfel flesh, Santana flesh**, determined by ITC. (A) Thermogram, (B) Michaelis-Menten diagram, and (C) calculated parameters.

When testing extracts from the flesh of Santana and Bohnapfel at equal polyphenol concentrations through different dilutions, Santana exhibited significantly weaker inhibition than Bohnapfel (Fig. 6). The weaker inhibition observed in Santana is likely attributable to its lower flavanol content, which amounts to 3 mg/100 g DW, comprising 2 mg/100 g DW of epicatechin (EC) and catechin (CAT) and 1 mg/100 g DW of di- and trimeric procyanidins (Kaeswurm et al. 2022 and 2023). In contrast, Bohnapfel contains higher levels of flavanols, 412 mg/100 g DW in total, which includes 99 mg/100 g DW EC and CAT, along with oligomeric procyanidins ranging from dimers to heptamers with 313 mg/100 g DW. Previous studies have reported that oligomeric procyanidins of high molecular weight exhibited a strong protein interaction (Soares et al., 2007) and highlight their potential as natural inhibitors of digestive enzymes (Gu et al., 2011; Liu et al., 2017). It is obvious that the inhibitory activity was not solely dependent on the total content of polyphenols, but also on the presence of specific subgroups of polyphenols such as oligomeric procyanidins.

### 3.3 Inhibitory effects of aronia extracts on *α*-amylase activity

**Fig. 7:**
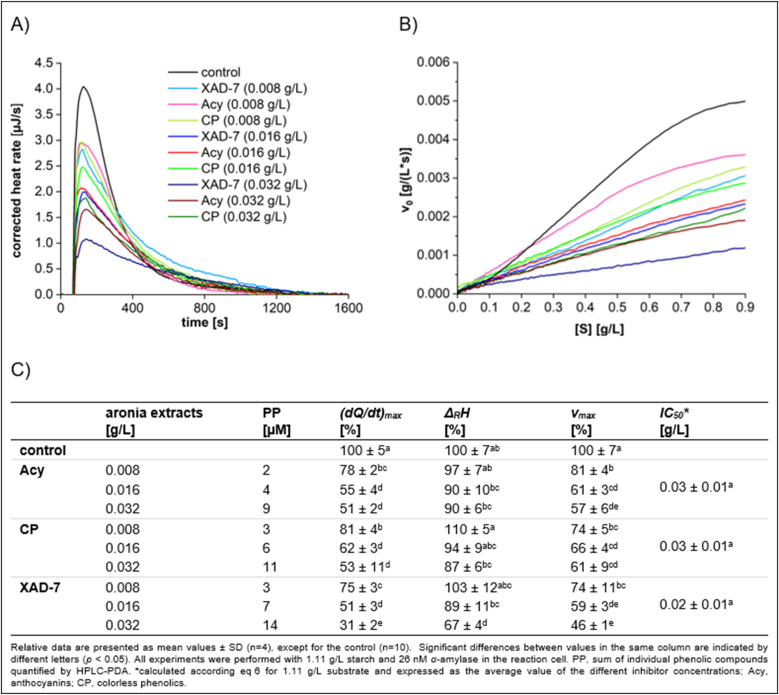
Characteristics of starch conversion by *α*-amylase incubated with **aronia** anthocyanins, colorless phenolics, complex XAD-7 extract, determined by ITC. (A) Thermogram, (B) Michaelis-Menten diagram, and (C) calculated parameters.

The complex aronia XAD-7 extract, and fractions containing the anthocyanins and colorless phenolics at different concentrations were investigated according to their inhibitory effects on *α*-amylase activity (Fig. 7). For all applied extracts and concentrations, a strong reduction in *α*-amylase activity was observed. However, disparities between the complex XAD-7 extract and the fractions of anthocyanins, as well as colorless phenolics, became evident in the reduction of the hydrolysis rate *(dQ/dt)*_*max*_. While *(dQ/dt)*_*max*_ was not effected at lower concentrations (0.008 g/L and 0.016 g/L), a notable decrease was observed for the complex XAD-7 extract at 0.032 g/L. At lower concentrations, no change in *Δ*_*R*_*H*_*app*_ was observed. This indicates that all extracts exhibited reversible inhibition. For the complex XAD-7 extract at 0.032 g/L, a significant decrease in *Δ*_*R*_*H*_*app*_ was observed, indicating an irreversible aggregation of *α*-amylase and polyphenols. These findings revealed a concentration-dependent inhibition of *α*-amylase by aronia phenolics but raised the question about the compounds being responsible for the superior inhibitory effect of the complex XAD-7 extract.

### 3.4 Inhibitory effects of tea extracts on *α*-amylase activity

**Fig. 8:**
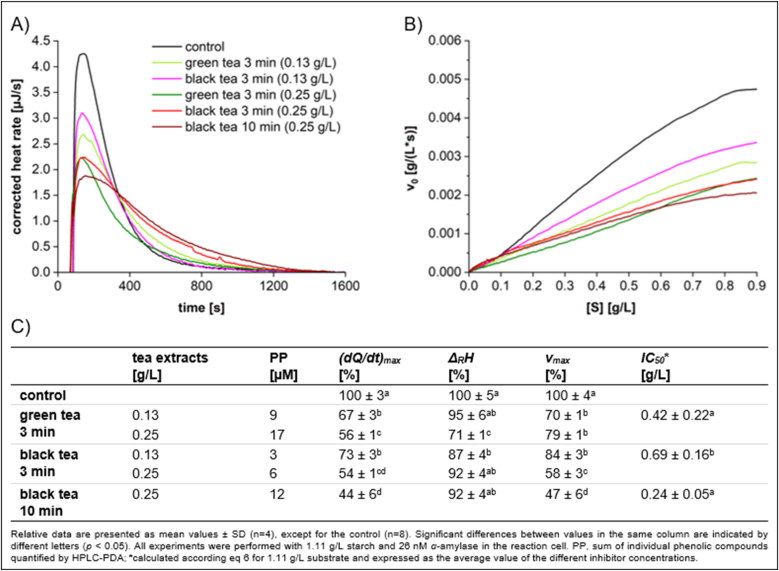
Characteristics of starch conversion by *α*-amylase incubated with **black tea and green tea** at different concentrations and steeping times, determined by ITC. (A) Thermogram, (B) Michaelis-Menten diagram, and (C) calculated parameters.

A clear trend of increased inhibition with longer steeping times is illustrated in Fig. 8, which was attributed to the increased phenolic content in extracts steeped for extended durations (S8). At a concentration of 0.13 g/L, green tea exhibited a slightly stronger inhibitory effect than black tea, obvious in *(dQ/dt)*_*max*_ and *v*_max_. The *Δ*_*R*_*H*_*app*_ values for both teas were similar to the control, indicating a reversible inhibition. At a concentration of 0.25 g/L, both teas revealed a generally higher inhibitory effect than at 0.13 g/L. However, a significant decrease in *Δ*_*R*_*H*_*app*_ was observed for green tea, suggesting the occurrence of irreversible aggregation between green tea polyphenols and *α*-amylase, whereas black tea displayed reversible inhibition. In conclusion, the data demonstrated that green tea exhibited a stronger inhibitory effect than black tea. The greater inhibitory effect was attributed to the higher phenolic content found in green tea (S8).

### 3.5 Inhibitory effect of black carrot extracts on *α*-amylase activity

**Fig. 9:**
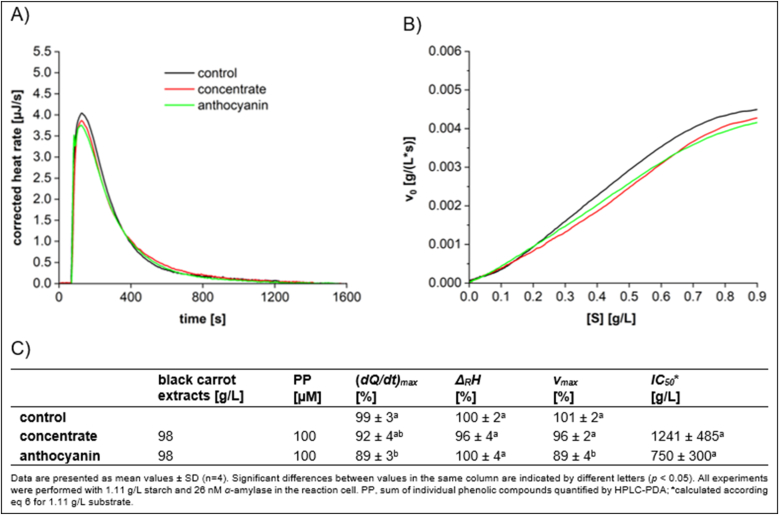
Characteristics of starch conversion by *α*-amylase incubated with black carrot concentrate and anthocyanin extract, determined by ITC. (A) Thermogram, (B) Michaelis-Menten diagram, and (C) calculated parameters.

To investigate the inhibitory effect of black carrot, both black carrot concentrate and its anthocyanin extract were studied. The results (Fig. 9) indicated that both the black carrot concentrate and the anthocyanin extract exhibited limited inhibition on *α*-amylase activity, with *(dQ/dt)*_*max*_ values of 92 % and 89 %, respectively. The nearly identical inhibitory effects suggested that the black carrot concentrate contained few non-anthocyanin phenolic compounds. In addition, for both the concentrate and the anthocyanin extract, a reversible inhibition was determined.

## 4 Discussion

### 4.1 Inhibitory effects of polyphenols on *α*-amylase activity

The *IC*_*50*_ values calculated are consistent with previous findings (Table 1) for most of the polyphenols. However, Li et al. (2009) and Souza Schmidt Gonçalves et al., (2010) indicated an *IC*_*50*_ value for rutin of less than 10 μM, which is in strong contrast to our findings (>1000 μM). In addition, they reported significantly lower *IC*_*50*_ for CA and Q-3-glc than our results. In both studies, starch hydrolysis was detected by UV/Vis spectroscopy after the reaction with 3,5-dinitrosalicylic acid (DNS). The discrepancy in *IC*_*50*_ values might be attributed to the interference of the reducing potential of polyphenols with the oxidation of the DNS by reducing sugars delivered by starch hydrolysis.

**Table 1:** *In vitro α*-amylase inhibition strengths and inhibition type of polyphenols reported for different assays.

PP	substrate	assay	*K*_*ic*_ [μM]	*K*_*iu*_ [μM]	inhibition type	*IC*_*50*_ [μM]	references
**FA**	G_2_-*p*NP	*p*NP UV/Vis	11170	4870	mixed	5450	Narita and Inouye (2011)
	starch	D-glucose assay UV/Vis	3404	3630	mixed	3203	Zheng et al. (2020)
	starch	DNS UV/Vis	N.A.	N.A.	N.A.	>5150	Aleixandre et al. (2022)
	starch	ITC	1237 ± 235	2358 ± 1478	mixed	2350 ± 1467	

**rutin**	starch	DNS UV/Vis	8.96	N.A.	competitive	N.A.	Li et al. (2009)
	starch	DNS UV/Vis	N.A.	N.A.	N.A.	1.2	Souza Schmidt Gonçalves et al., (2010)
	starch	ITC	354 ± 55	1562 ± 872	mixed	1545 ± 855	

**PHL**	GalG_2_CNP	CNP UV/Vis	151	391	mixed	391	Kaeswurm et al. (2019)
	starch	PAHBAH UV/Vis	–	–	–	–	Li et al. (2019)
	starch	ITC	418 ± 127	573 ± 145	mixed	572 ± 145	

**CA**	starch	DNS UV/Vis	N.A.	N.A.	N.A.	3.9	Souza Schmidt Gonçalves et al. (2010)
	GalG_2_CNP	CNP UV/Vis	406	413	mixed	413	Kaeswurm et al. (2019)
	G_2-_*p*NP	*p*NP UV/Vis	1120	270	mixed	400	Narita and Inouye (2011)
	starch	ITC	195 ± 68	279 ± 100	mixed	278 ± 100	

**CYD-3-glc**	starch	PAHBAH UV/Vis	390	210	mixed	340	Le et al. (2024)
	starch	DNS UV/Vis	N.A.	N.A.	N.A.	300	Akkarachiyasit et al., (2010)
	starch	DNS UV/Vis	140	N.A.	competitive	240	Sui et al. (2016)
	starch	ITC	143 ± 30	201 ± 34	mixed	200 ± 34	

**Q-3-glc**	starch	DNS UV/Vis	5.35	N.A.	competitive	N.A.	Li et al. (2009)
	starch	ITC	115 ± 16	179 ± 28	mixed	178 ± 28	

**EC**_ox_	GalG_2_CNP	CNP UV/Vis	48	241	mixed	241	Kaeswurm et al. (2019)
	starch	ITC	77 ± 18	111 ± 17	mixed	111 ± 17	

**EGCG**	amylose, amylopectin	DNS UV/Vis	N.A.	N.A.	N.A.	5.3–60	Nyambe-Silavwe et al. (2015)
	GalG_2_CNP	CNP UV/Vis	124	58	mixed	58	Kaeswurm et al. (2019)
	starch	ITC	19 ± 2	26 ± 2	mixed	25 ± 2	

N.A.: No data available; “–” no inhibition; GalG_2_CNP, chloronitrophenyl-galactopyranosylmaltoside; CNP, chloronitrophenol; PAHBAH, 4-hydroxybenzoic acid hydrazide; DNS, 3,5-dinitrosalicylic acid; *p*NP, *p*-nitrophenol; G_2_-*p*NP, *p*-nitrophenyl-α-D-maltoside. The IC_50_ values are determined based on the substrate concentration used, which varies across different experiments.

Based on the data in our study, some differences in the inhibition mechanisms are observed. While a pure mixed inhibition identified pattern for FA is consistent with the mechanism published by Zheng et al. (2020), who also utilized starch as the substrate, Narita et al. reported for the substrate G_2_-*p*NP a mixed inhibition with a predominantly uncompetitive portion (Narita and Inouye, 2011). Thus, the substrate seems to have a significant effect on the determination of the inhibition mechanism.

Discrepancies in inhibition values and mechanisms among studies are likely due to different assays, substrates and detection methods. This is most pronounced when a comparison is made with the data set provided by Kaeswurm et al., (2019). This study used identical phenolics and *α*-amylase, however applied an assay based on the artificial substrate chloronitrophenyl-galactopyranosylmaltoside (GalG_2_CNP). In addition, inhibition by oxidized EC may be influenced by the non-reproducible formation of browning products, which can affect inhibition outcomes. Variations in substrate concentrations, as well as a prior incubation of polyphenols with the *α*-amylase or the substrate, might significantly affect the *IC*_*50*_ measurements (Nyambe-Silavwe et al., 2015). Therefore, it is crucial to compare inhibitory effects of phenolics under standardized conditions. Using experimental conditions that closely mimic physiological environments would further increase the relevance and applicability of the results.

### 4.2 Inhibitory effects of apple extracts on *α*-amylase activity

The primary phenolic components found in the flesh of apples include flavanols (50–79 %, except for Santana with 3 %), hydroxycinnamic acid derivatives (16–82 %), and chalcone glycosides (3–14 %) (Kaeswurm et al., 2022, 2023). Among the chalcones, phlorizin is the predominant phenolic compound in apples; however, due to their weak inhibitory effects (Fig. 2), chalcones are unlikely to be a significant contributor to the inhibitory activity of apple flesh extracts. Chlorogenic acid, the major polyphenol among the hydroxycinnamic acid derivatives, demonstrates moderate inhibition (Fig. 2) and thus may potentially contribute to the overall inhibitory effects of apple flesh extracts. However, the variety Santana, which contains 82 % hydroxycinnamic acid derivatives in the apple flesh, exhibits very weak inhibitory effect, suggesting that these compounds may not significantly influence the overall inhibitory effects. Apples contain the monomeric flavanols catechin and epicatechin as well as procyanidins, ranging from dimers to octamers. Claasen et al. revealed that procyanidins exhibited significantly inhibitory effects (Claasen et al., 2025). Thus, Bohnapfel, which contains markedly amounts of oligomeric procyanidins, exerts the most effective *α*-amylase inhibition among the apples investigated. This indicates that oligomeric procyanidins are key compounds responsible for the inhibitory activity observed in apple flesh extracts.

Apple peel demonstrates an even stronger inhibitory effect than the flesh. This is attributed to the increased phenolic content in peel, which is approximately two to three times higher than found in the flesh. Flavonols are predominantly present in peels, accounting for 21–37 %, with quercetin derivatives being the major structures. Anthocyanins make up 4–5 % of all phenolics in peels of red-skinned apple varieties, including Santana, Gewürzluiken, and Bohnapfel, with cyanidin-3-*O*-glucoside identified as the primary anthocyanin. Both phenolic structures exhibit moderate *α*-amylase inhibitory activity (Fig. 2), and their additional presence in the peel may further contribute to the enhanced inhibitory effects observed in the peel.

Only a few studies have investigated the inhibitory effects of apple extracts. Zhao et al. (2019) examined five cultivars that differ from those in our study, reporting *IC*_*50*_ values ranging from 0.023 to 0.541 g/L. In our research, we observe slightly higher *IC_50_* values within the following ranges: for the peel, due to the different varieties, *IC*_*50*_ varies from 0.36 to 4.30 g/L, and for the flesh, from 0.84 to 5.03 g/L. It should be noted that both the differences in the apple varieties investigated and the assay methods used to assess inhibition contribute to the difference observed between our results and those of previous studies. The *IC*_*50*_ value for Santana flesh could not be determined. This is because the observed approximately 7 % acceleration of *v*_max_ in Santana flesh results in a negative *K*_*iu*_ value, making the *IC*_*50*_ calculation impossible (see eqs (5) and (6)). It is important to note that there is no evidence suggesting polyphenols accelerate the conversion rate of *α*-amylase. Therefore, the apparent 7 % increase in conversion rate in Santana flesh is likely attributable to fluctuations in *α*-amylase concentration.

### 4.3 Inhibitory effects of aronia extracts on *α*-amylase activity

The colorless phenolic fraction (S6) primarily consists of neochlorogenic acid (48 %) and chlorogenic acid (50 %). The anthocyanin fraction is predominantly composed of cyanidin-3-*O*-galactoside (68 %) and cyanidin-3-*O*-arabinoside (28 %). Cyanidin-3-*O*-glucoside and cyanidin-3-*O*-xyloside are only present in minor amounts. The anthocyanin extract demonstrates an inhibitory effect similar to that of the colorless phenolic extracts (Fig. 7), which aligns with the similar inhibitory effects observed for standard compounds chlorogenic acid and cyanidin-3-*O*-glucoside (Fig. 2). Notably, the aronia XAD-7 extract demonstrates stronger inhibitory activity than both fractions obtained from it. This might be due to the presence of procyanidins with molecular weights exceeding tetramers markedly enhancing *α*-amylase inhibition (Rodríguez-Werner et al., 2019; Kostka et al., 2020). Identification and quantification of these compounds are challenging due to adsorption on cell wall polysaccharides and the presence of various binding isomers. Furthermore, during separation of the anthocyanin and colorless phenolics fraction, they might be adsorbed onto the membrane. These high molecular compounds significantly influence *α*-amylase inhibition and are suspected to be the primary contributors to the observed inhibitory effect in the aronia XAD-7 extract (Claasen et al., 2025; Wang et al., 2020). Moreover, while chlorogenic acid and cyanidin-3-*O*-glucoside are recognized as reversible inhibitors (Fig. 2), the significant decrease in *Δ*_*R*_*H*_*app*_ at a concentration of 0.032 g/L for the aronia XAD-7 extract further suggests that oligomeric procyanidins are the main inhibitory agents. Probably, through irreversible aggregation oligomeric procyanidins play a more prominent role in *α*-amylase inhibition. Few studies have specifically examined the inhibitory effects of aronia extracts, demonstrating marked differences. For example, Berger et al. (2020) reported a strong *α*-amylase inhibition (*IC*_*5*0_ = 0.33 ± 0.08 g/L), while Worsztynowicz et al. (2014) found a substantially higher *IC*_*50*_ value of 10.31 ± 0.04 g/L. Our data (*IC*_*50*_ 0.02–0.03 g/L) highlight the substantial inhibitory potential of aronia berries. However, due to very low inhibitor concentration applied in our experiments (to avoid the formation of aggregates), no significant differences in *IC*_*50*_ values were observed among the different extracts. Variations in the procyanidin levels due to adsorption onto cell wall material (trester) and technical membranes might explain the discrepancies between the literature data. Furthermore, procyanidin concentrations may fluctuate significantly depending on the processing method, which in turn affects the inhibitory efficacy of aronia products markedly.

### 4.4 Inhibitory effects of tea extracts on *α*-amylase activity

Black tea is produced from green tea through a complete fermentation process. During this fermentation, a significant portion of catechins is polymerized by polyphenol oxidase, resulting in the formation of theaflavins and other high molecular weight compounds (Sun et al., 2016a). Key polyphenols of green tea are EGCG, epicatechin-3-gallate, and EC (S8). Caffeine was also present in all sample extracts. However, the minor alterations in the caffeine content between green and black tea with the same steeping time indicate that caffeine is unlikely to be a significant factor responsible for the observed differences in inhibitory activity. While EC exhibits only moderate inhibitory effects (Fig. 2), EGCG demonstrates the most potent inhibition among the phenolic compounds, indicating its significant contribution to the overall inhibitory effect of green tea. Furthermore, black tea steeped for 10 min, exhibited a stronger inhibitory effect than green tea steeped for just 3 min, containing a significantly higher concentration of caffeine, a slightly elevated level of EGCG and a reduced concentration of epicatechin-3-gallate (S8). This observation reinforces the assertion that EGCG is the primary contributor to the inhibitory activity in tea. Most probably, the higher caffeine content in black tea also contributes to the overall inhibitory effect (Rajan, 2018). It is noted that due to the fermentation process, black tea has a lower concentration of polyphenols, with decreased levels of EGCG and a reduction in epicatechin-3-gallate, while no EC is detected (S8). The fermentation process produces high molecular weight compounds (browning products), which exhibit strong inhibitory effects (see EC_ox_, Fig. 2). However, due to their hydrophobic nature, the solubility in polar aqueous solutions is limited, and thus the extraction of these compounds during the steeping process is limited. As a result, the inhibitory effect of these high molecular weight browning products may not significantly contribute to the overall inhibition observed in black tea.

Sun et al. (2016a) reported that green tea had a stronger inhibitory effect (*IC*_*50*_ = 0.20 g/L) than black tea (*IC*_*50*_ = 0.46 g/L) due to its higher total phenolic content at a steeping time of 40 min. In our study, when steeped for 10 min, black tea exhibits an *IC*_*50*_ of 0.24 g/L, which aligns closely with the results reported by Sun et al. With a shorter steeping time of 3 min, the inhibitory effect of black tea decreased, with an *IC*_*50*_ value of 0.69 g/L. Consistent with the findings of Sun et al., green tea demonstrated a slightly better inhibitory effect (*IC*_*50*_ = 0.42 g/L) than black tea.

### 4.5 Inhibitory effects of black carrot on *α*-amylase activity

CYD-3-*O*-glc exhibits a significantly stronger inhibition of *α*-amylase activity than black carrot concentrate and its anthocyanin fraction (Fig. 2, Fig. 9). This confirms that the acylated form present in black carrot extract is less effective than the non-acylated form. This weaker inhibition observed with acylated anthocyanins relative to their non-acylated counterparts may be due to the two key factors. 1.) Steric hindrance: The acylation of the sugar moiety with aromatic acids leads to formation of a sandwich-type complex known as intramolecular co-pigmentation (Giusti and Wrolstad, 2003). This increases the steric bulk of the molecule, preventing the anthocyanin from covering the enzyme's surface. 2.) Reduced substrate Interaction: The acyl group may alter interaction of the anthocyanin with the substrate, and thus the hydrolysis by *α*-amylase. Our findings contrast with those reported by Kaeswurm et al. (2020), who found a stronger inhibition of *α*-amylase by acylated anthocyanins. In our study, the inhibitory effect was very weak, with *IC*_*50*_ values exceeding 750 g/L. Due to differences in anthocyanin quantification (UV spectroscopy/ absorption coefficient vs. chromatography/ calibration curve, it is likely that the anthocyanin concentration in this study is overestimated.

### 4.6 Inhibitory effects of plant extracts and the complexity of structural specificity

The calculation of the inhibition parameter for the standard compounds based on mass concentration (S15) should provide a valuable support to evaluate the inhibitory effect of the extracts, where calculation based on molar concentration is not a viable option. The inhibition observed for the extract is a sum parameter due to the mixture of different phenolic structures and their individual contributions to inhibition strength and mechanism. The calculated values for standard compounds further illustrate the substantial impact of the molecular weight, which is most apparent in the elevated values for *K*_*ic*_, *K*_*iu*_ and *IC*_*50*_ observed for PC C1, attributable to its higher molecular weight. Among the plant extracts evaluated, aronia exhibits the highest inhibitory activity, with *IC*_*50*_ values between 0.02 and 0.03 g/L. Tea limits *IC*_*50*_ values to 0.24–0.69 g/L, and apple extracts significantly vary in their inhibition, with *IC*_*50*_ values ranging from 0.36 to 5.03 g/L, depending on the specific apple variety. Black carrot extracts exhibit the weakest inhibitory activity, with *IC*_*50*_ values exceeding 750 g/L. Notably, apple, tea, and aronia extracts demonstrate significantly stronger inhibitory effects (*(dQ/dt)*_*max*_) at much lower polyphenol concentrations than the respective major standard compound (Fig. 2). For aronia extract, where sugars have been removed, the inhibitor concentration does not represent the actual inhibitory effect of a juice. This is analogous to the black carrot extract. Nevertheless, in contrast to all other extracts investigated, its inhibitory effect was substantially reduced relative to the standard compound CYD-3-glc.

It should be noted that to accurately determine *IC*_*50*_, the inhibition must remain within the reversible range, allowing for measurable *K*_*m*_ and **v**_*max*_ values (see eqs (1)–(6)). The concentrations of extracts used to maintain this reversible range are much lower than those encountered in real-world conditions. Additionally, precise measurement of the extract's mass concentration is essential for reliable *IC*_*50*_ calculation. However, the remarkable inhibitory capacity of most plant extracts may be attributed to the synergistic interactions among their individual compounds (Akkarachiyasit et al., 2010; Ni et al., 2024). Furthermore, oligomeric procyanidins, which significantly inhibit *α*-amylase (Claasen et al., 2025; Wang et al., 2020; Sun and Miao, 2020), are generally underestimated due to identification and quantification limitations (Lin et al., 2014). However, they might significantly contribute to the inhibition process. Generally, *in vitro* inhibition experiments are strongly dependent on phenolic concentration applied (Ni et al., 2024; Yang and Kong, 2016). Thus, methodological differences in polyphenol quantification might lead to discrepancies in the observed inhibition.

In conclusion, this study presents a comprehensive investigation into the inhibitory effects of polyphenols and polyphenol-rich extracts on *α*-amylase activity, using isothermal titration calorimetry to monitor starch digestion in real time. The data emphasize the critical role of the specific structural characteristics of each polyphenol in *α*-amylase inhibition, providing valuable insights into effective plant-based inhibitors to prevent the development of hyperglycemia and related metabolic disorders. However, it should be noted that *in vitro* analyses primarily elucidate fundamental structure-activity relationships. Their applicability to *in vivo* scenarios, particularly with regard to their impact on glycaemic control, requires validation through human studies. Additionally, phenolic extracts from aronia berries and black carrot were isolated to evaluate the inhibitory effects of the polyphenols individually, without taking into account the potential influence of other dietary components. For apples, which also contain terpenes and carotenoids (Acquavia et al., 2021), the dominant role of polyphenols for digestive enzyme inhibition has been highlighted in previous studies (Sun et al., 2016b; Li et al., 2019; Ercan and El, 2021; Sugiyama et al., 2007). However, the specific contribution of terpenes and carotenoids in apples to this effect has not been documented. For tea, a synergistic effect of L-theanine (Hao et al., 2025) and caffeine (Rajan, 2018) with polyphenols on *α*-amylase inhibition has been suggested, however further research is required to elucidate the complex interactions between phytochemicals which might enhance inhibitory responses. Moreover, further investigation is required into the impact of these polyphenols and extracts on other digestive enzymes.

## Data availability statement

The data generated during the study are included in this published article and its supplementary files.

## Author contributions

Experiments were performed by M.X. and J.P. Data analysis and curation were carried out by M.X. and discussed with M.B. The manuscript was written by M.X. and M.B. T.E. supported in resources, visualization and manuscript review and editing. M.B. conceptualized and supervised the study and was responsible for project administration and funding acquisition. All authors have given approval to the final version of the manuscript.

## Funding

This research was funded by the Dr. Leni Schöninger Foundation, GermanyFunds of the Chemical Industry, Germany (10.13039/100018992FCI) and financial support of the 12-month position of M.X. by the faculty of chemistry at the 10.13039/501100009534University of Stuttgart, Germany.

## Declaration of competing interest

The authors declare that they have no known competing financial interests or personal relationships that could have appeared to influence the work reported in this paper.
