# Supplementary material for: Structure-effect relationship of phenolic compounds on α-amylase inhibition studied by isothermal titration calorimetry
Source: Curr Res Food Sci. 2025 Dec 6;12:101266. doi: 10.1016/j.crfs.2025.101266 (PMC12810351; doi:10.1016/j.crfs.2025.101266)
Supplement: Multimedia component 1 [file mmc1.pdf]

Supplemental Material

Structure-Effect Relationship of Phenolic Compounds on α-Amylase Inhibition Studied by Isothermal Titration Calorimetry

Mengyao Xiong[1], Jörn Plambeck[1], Tuba Esatbeyoglu[2], and Maria Buchweitz[1]*

[1]Department of Chemistry, Institute of Food Chemistry, University of Hamburg, 20146 Hamburg, Germany.

[2]Department of Molecular Food Chemistry and Food Development, Institute of Food and One Health, Leibniz University of Hannover, 30167 Hannover, Germany.

*Corresponding author: M. Buchweitz

Phone: +49 40 42838 7979; Email: maria.buchweitz@uni-hamburg.de

---

Apple polyphenols

S1: Phenolic content [µg/L extract] and profile [% of total phenolics] in flesh of different apple varieties.[1]

variety	PP [µM] in extract	PP [µg/L extract]	flavonols [µg/L extract]	flavanols [µg/L extract]	chalcone glycosides [µg/L extract]	hydroxycinnamic acid derivates [µg/L extract]
**Golden Delicious**	2394 ± 99	608 ± 12	8 ± 0 (1%)	324 ± 4 (53%)	56 ± 0 (9%)	220 ± 4 (36%)
**Santana**	2229 ± 27	448 ± 8	0 ± 0 (0%)	12 ± 0 (3%)	64 ± 0 (14%)	368 ± 4 (82%)
**Granny Smith**	2387 ± 3	656 ± 4	4 ± 0 (1%)	516 ± 4 (79%)	32 ± 0 (5%)	104 ± 0 (16%)
**Gewürzlu-iken**	10214 ± 433	2416 ± 100	8 ± 0 (0%)	1220 ± 44 (50%)	80 ± 8 (3%)	1104 ± 44 (46%)
**Bohnapfel**	9889 ± 479	2580 ± 64	12 ± 0 (0%)	1648 ± 48 (64%)	184 ± 0 (7%)	736 ± 16 (29%)

[1]The data were calculated based on Kaeswurm et al. (Kaeswurm et al., 2022; Kaeswurm et al., 2023); PP, sum of all polyphenols quantified by HPLC-PDA. The percentage (%) was calculated from the sum of individual compounds relating to the total sum of phenolics.

S2: Phenolic content [µg/L extract] and profile [% of total phenolics] in peel of different apple varieties.[1]

variety	PP [µM] in extract	PP [µg/L extract]	flavonols [µg/L extract]	flavanols [µg/L extract]	chalcone glycosides [µg/L extract]	hydroxycinnamic acid derivates [µg/L extract]	antho-cyanins [µg/L extract]
**Golden Delicious**	2094 ± 40	1732 ± 32	356 ± 4 (21%)	1016 ± 20 (59%)	208 ± 4 (12%)	152 ± 4 (9%)	n.q.
**Santana**	1982 ± 15	1512 ± 20	508 ± 8 (34%)	508 ± 8 (34%)	216 ± 0 (14%)	212 ± 4 (14%)	68 ± 0 (4%)
**Granny Smith**	2186 ± 153	1844 ± 136	684 ± 48 (37%)	1012 ± 76 (55%)	132 ± 12 (7%)	16 ± 0 (1%)	n.q.
**Gewürzlu-iken**	5804 ± 227	4608 ± 196	1196 ± 52 (26%)	2256 ± 104 (49%)	220 ± 8 (5%)	692 ± 20 (15%)	244 ± 12 (5%)
**Bohnapfel**	9641 ± 126	7800 ± 132	2920 ± 80 (37%)	3636 ± 12 (47%)	812 ± 24 (10%)	412 ± 4 (5%)	20 ± 0 (0%)

[1]The data were calculated based on Kaeswurm et al. (Kaeswurm et al., 2022; Kaeswurm et al., 2023); n.q.: not quantifiable; PP, sum of all polyphenols quantified by HPLC-PDA. The percentage (%) was calculated from the sum of individual compounds relating to the total sum of phenolics.

Aronia polyphenols

The analysis of the different fractions was performed with modifications based on Rodríguez-Werner et al. (Rodríguez-Werner et al., 2019), using an Agilent 1200 Series HPLC system equipped with a G1315D PDA detector, G1312A binary pump, G1316A column oven, G1329A autosampler and a G7122A 1260 Series degasser. A Luna C18, 5 µm, 125 Å, 250 x 4.0 mm column with a guard column (Phenomenex, Aschaffenburg, Germany) was used as stationary phase. Mobile phases consisted of solvent A (water/acetonitrile/acetic acid, 87:3:10 *v/v/v*) and solvent B (water/acetonitrile/acetic acid, 40:50:10 *v/v/v*). The elution was carried out on gradient mode with a flow rate of 0.5 mL/min: 3 – 20% B (0 – 65 min), 20 – 40% B (65 – 75 min), 40 – 60% B (75 – 80 min), 60% – 90% B (80 – 85 min). Afterwards, a re-equilibration for a period of 6 min was conducted. Detection takes place at λ = 280, 320, 520 nm with a column temperature of 25 °C. Quantification was performed for the phenolic acids as chlorogenic acid equivalents (0.77 to 245.75 mg/L; $R^2 > 0.999$, y = 133781x –

78.678) at *λ* = 320 nm by external calibration. Anthocyanins were monitored at *λ* = 520 nm and quantified as cyanidin-3-galactoside (0.72 to 231.33 mg/L; R^2 > 0.999, y = 148750x + 137.91). Biological triplicates and technical duplicates were performed for all HPLC measurements.

S3: HPLC-PDA chromatograms of the complex XAD-7 extract recorded at *λ* = 280 nm (A), 320 nm (B) and 520 nm (C).

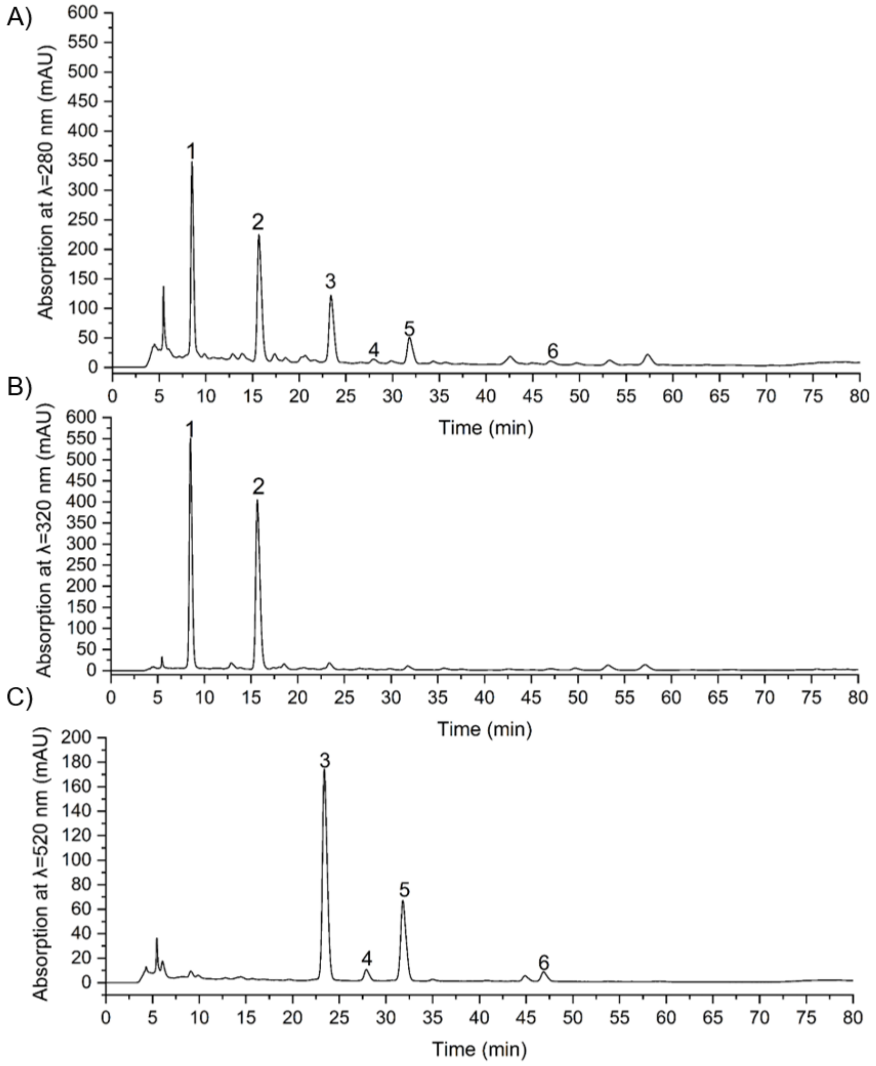

S4: Quantification of the XAD-7 extract of *Aronia melanocarpa* determined by HPLC-PDA.[1]

peak	compound	absorption maxima [nm]	content [mg/g]	content [µmol/g]
**1**	neochlorogenic acid	324	58.96 ± 1.67	166.41 ± 4.71
**2**	chlorogenic acid	324	63.37 ± 1.45	178.85 ± 4.09
**3**	cyanidin-3-*O*-galactoside	516	27.59 ± 0.30	56.91 ± 0.62
**4**	cyanidin-3-*O*-glucoside	517	0.97 ± 0.01	2.00 ± 0.02
**5**	cyanidin-3-*O*-arabinoside	519	11.28 ± 0.13	24.80 ± 0.29
**6**	cyanidin-3-*O*-xyloside	518	0.96 ± 0.02	2.11 ± 0.04
	**sum**		163.13	431.09

Data are presented as mean values ± SD (n=6); [1]quantification was performed by external calibration using standard compounds.

S5: HPLC-PDA chromatograms of the colorless phenolic fraction recorded at λ = 320 nm (A) and of the anthocyanin fraction at λ = 520 nm (B).

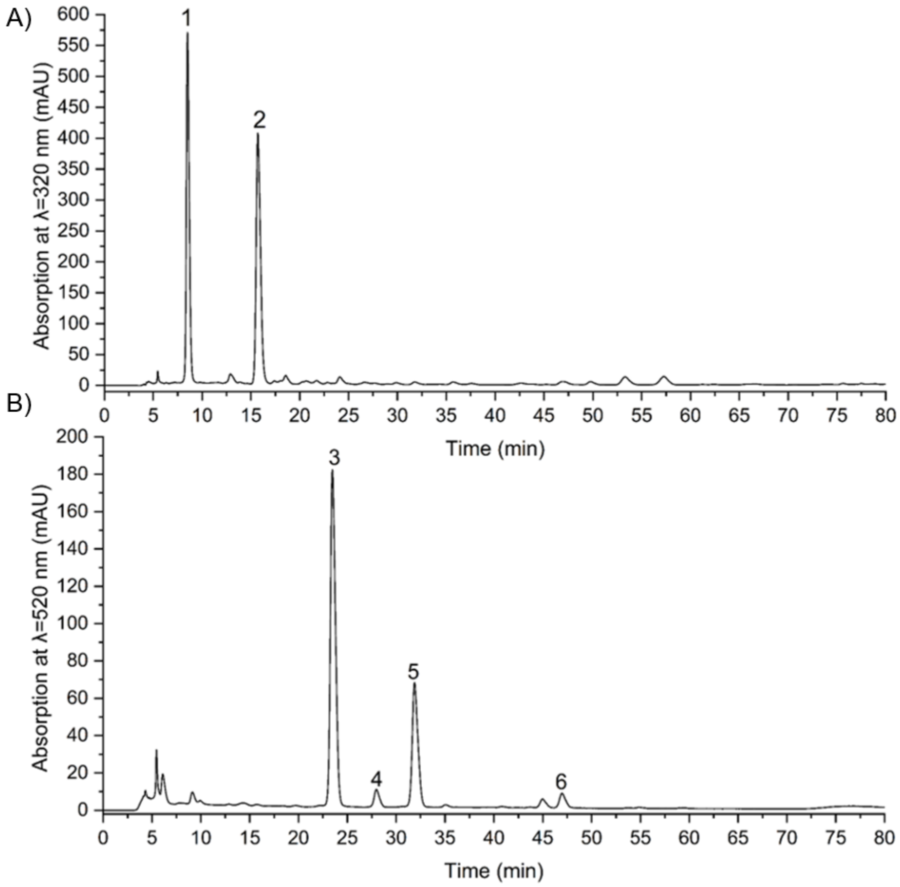

S6: Quantification of the colorless phenolic and anthocyanin fractions of *Aronia melanocarpa* eluated from XAD-7 resin analyzed by HPLC-PDA.[1]

peak	compound	colorless phenolic content [mg/g]	colorless phenolic content [µmol/g]	anthocyanin content [mg/g]	anthocyanin content [µmol/g]
1	neochlorogenic acid	59.77 ± 0.62	168.69 ± 1.75	n.q.	n.q.
2	chlorogenic acid	62.77 ± 0.49	177.16 ± 1.38	n.q.	n.q.
3	cyanidin-3-*O*-galactoside	2.07 ± 0.15	4.27 ± 0.31	85.91 ± 3.14	177.21 ± 6.48
4	cyanidin-3-*O*-glucoside	n.q.	n.q.	3.03 ± 0.17	6.25 ± 0.35
5	cyanidin-3-*O*-arabinoside	0.59 ± 0.00	1.30 ± 0.00	34.77 ± 1.23	76.45 ± 2.70
6	cyanidin-3-*O*-xyloside	n.q.	n.q.	2.83 ± 0.15	6.22 ± 0.33
	**sum**	125.20	351.42	126.54	266.13

Data are presented as mean values ± SD (n=6); n.q.: not quantifiable; [1]quantification was performed by external calibration using standard compounds.

Tea polyphenols

The separation of polyphenols from tea for characterization and quantification was performed using an Agilent Infinity 1260 HPLC system (Agilent, Santa Clara, USA), which featured a binary pump (1260 ALS) including an autosampler, which was cooled to 10 °C, and a degasser. The system was equipped with a C18 Nucleodur Gravity-SB column (150 × 2 mm, ID 2 µm (Macherey Nagel, Düren, Germany). The column oven was maintained at 35 °C, with a flow rate of 0.25 mL/min. Tea extracts were diluted 1:4 in 0.1 M phosphate buffer (pH 7.0) containing 0.4 M NaCl and then a volume of 5 µL was injected for analysis. Eluent A consisted of a mixture of formic acid, acetonitrile, and water in a

ratio of 1:2:97 (*v/v/v*), while eluent B contained formic acid and acetonitrile in a ratio of 1:99 (*v/v*). The initial conditions were set to 94% eluent A and 6% eluent B. Eluent B was increased to 33% in 45 min, followed by a further increase to 55% over 52 min. The column was washed with 100% B for 2 min, then the gradient returned to the initial conditions in 58 min and the system was allowed to re-equilibrate for a further 9 min. The individual concentrations of compounds were determined using their respective external calibration, with the area (y) expressed in [mAU*s] and concentration (x) in [mg/L]: caffeine (10.00 to 70.00 mg/L, $R^2 > 0.999$, y = 47.645x), (-)-epicatechin (8.41 to 84.12 mg/L, $R^2 > 0.999$, y = 13.049x), epigallocatechin gallate (21.36 to 149.52 mg/L, $R^2 > 0.999$, y = 22.654x), and epicatechin gallate (3.04 to 30.40 mg/L, $R^2 > 0.999$, y = 25.227x). Triplicates were performed for the HPLC measurements.

S7: HPLC-PDA chromatograms recorded at λ = 280 nm for tea extracts prepared at 2.5 g/L: (A) green tea steeped for 3 min; (B) black tea steeped for 3 min; (C) black tea steeped for 10 min.

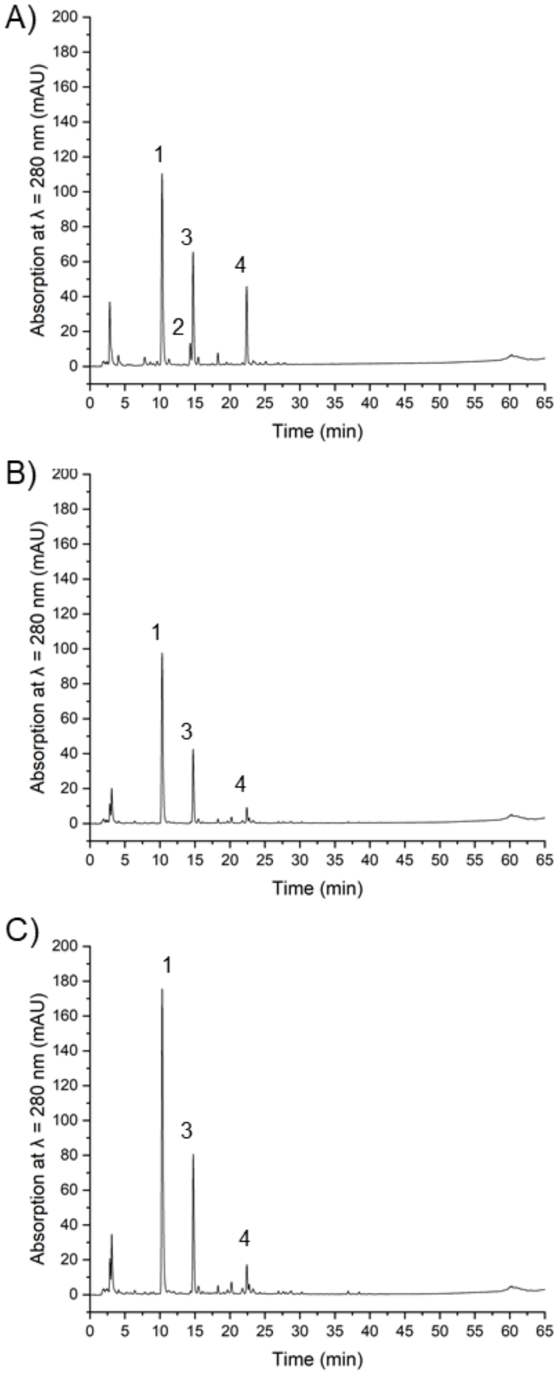

S8: Quantification of compounds in green tea and black tea determined by HPLC-PDA.[1]

peak	compound	green tea 3 min content [mg/g]	green tea 3 min content [µmol/g]	black tea 3 min content [mg/g]	black tea 3 min content [µmol/g]	black tea 10 min content [mg/g]	black tea 10 min content [µmol/g]
**1**	caffeine	12.99 ± 0.14	66.88 ± 0.71	11.50 ± 0.24	59.24 ± 1.26	21.37 ± 0.05	110.07 ± 0.27
**2**	EC	4.38 ± 0.10	15.11 ± 0.34	n.q.	n.q.	n.q.	n.q.
**3**	EGCG	15.02 ± 0.32	32.78 ± 0.69	9.09 ± 0.77	19.83 ± 1.68	18.47 ± 0.43	40.30 ± 0.95
**4**	epicatechin-3-gallate	8.52 ± 0.11	19.27 ± 0.25	1.59 ± 0.06	3.59 ± 0.13	3.03 ± 0.03	6.84 ± 0.07
	**sum[2]**	27.93	67.15	10.68	23.42	21.50	47.15

Data are presented as mean values ± SD (n=3); n.q.: not quantifiable; [1]quantification was performed by external calibration using standard compounds; [2]sum of phenolic compounds including EC, EGCG, epicatechin-3-gallate.

Black carrot polyphenols

Both the black carrot concentrate and anthocyanin extract (1,247 g/L) were diluted 1:8 in acidified water (0.01% HCl) and subsequently analyzed by HPLC-PDA, following the method outlined by Kaeswurm et al. (Julia A. H. Kaeswurm et al., 2020). Anthocyanins were quantified at 520 nm, using an external calibration of cyanidin-3-*O*-glucoside (9.83 to 491.60 mg/L, $R^2 > 0.999$, $y = 73.163x$), with the area (y) expressed in [mAU*s] and concentration (x) in [mg/L]. Duplicate measurements were performed for the HPLC analysis.

S9: HPLC-PDA chromatograms recorded at λ= 520 nm for (A) anthocyanin extract; (B) black carrot concentrate

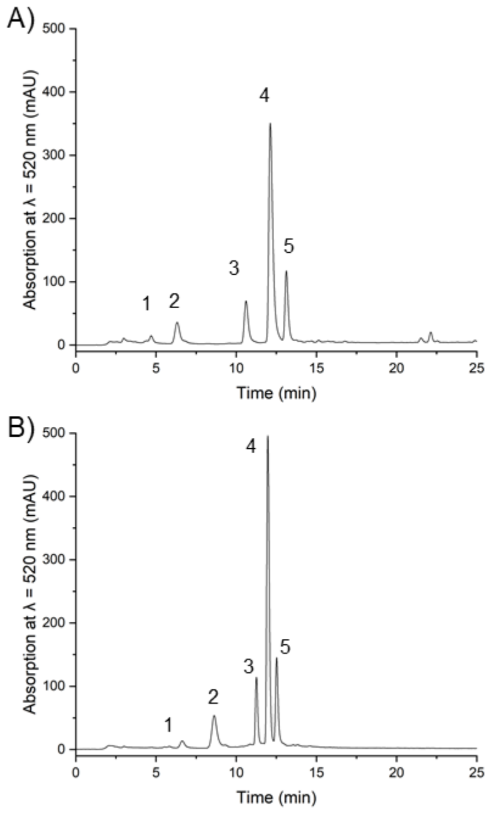

S10: Quantification of anthocyanins in black carrot concentrate and anthocyanin extract by HPLC-PDA.[1]

peak	anthocyanins	concentrate mass content [mg/L]	concentrate concentration [µM]	anthocyanin extract mass content [mg/L]	anthocyanin extract concentration [µM]
1	cyd-3-gal-xyl-glc	26.55 ± 0.41	35.78 ± 0.55	22.10 ± 0.24	29.79 ± 0.32
2	cyd-3-gal-xyl	138.91 ± 6.80	239.50 ± 11.73	94.55 ± 6.63	163.02 ± 11.43
3	cyd-3-gal-xyl-glc	125.27 ± 5.29	132.14 ± 5.58	131.57 ± 2.71	138.79 ± 2.86
4	cyd-3-gal-xyl-glc	605.47 ± 3.46	659.56 ± 3.77	656.36 ± 34.24	714.99 ± 37.30
5	cyd-3-gal-xyl-glc	182.35 ± 1.70	205.35 ± 1.91	181.22 ± 3.74	204.08 ± 4.21
	**sum**	1078.55	1272.33	1085.82	1250.67

Data are presented as mean values ± SD (n=2); [1]quantification was performed by external calibration using standard compounds.

S11: Optimization of the ITC assay to achieve maximal enzyme saturation with minimal product inhibition conducted by varying the injection volume of starch (40 µg/µL) into 26 nM *α*-amylase.

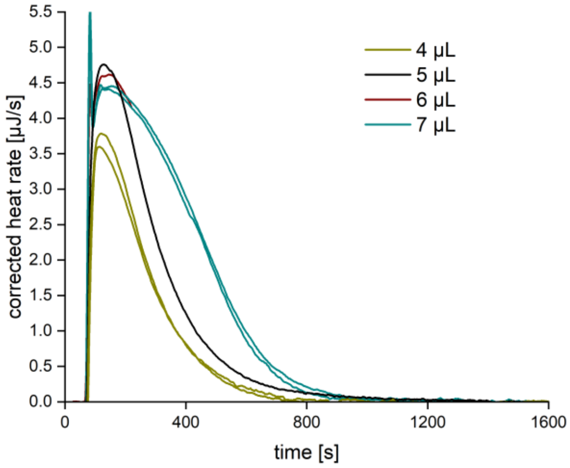

S12: Correlation of the IC_{50} values with the total molecular weight (▲), the molecular weight of the aglycon (●), and the molecular weight of the atoms contributing to the conjugated system (■) for the different phenolic structures. The expansion of the conjugated system has been roughly estimated by the number of sp^2 carbons (FA, 9; Rutin, 15; PHL, 13; CAT, 12; EC, 12; CA, 9; PC B1, 24; PC B2, 24; CYD-3-glc, 15; Q-3-glc, 15; EGCG, 19; PC C1, 36) multiplied by the molecular weight of carbon (12 g/mol).

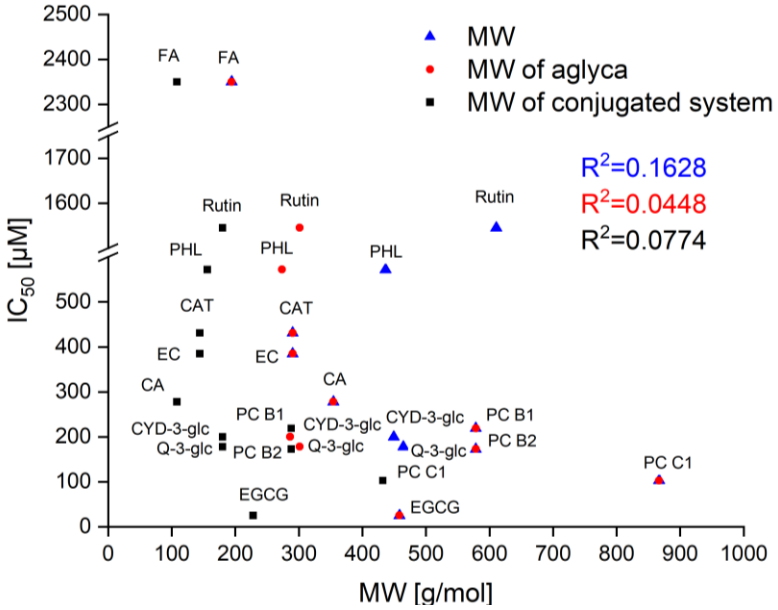

S13: Correlation of *(dQ/dt)max* [%] with polyphenol concentration [µM] (A) and $\Delta_R H_{app}$ [%] with polyphenol concentration [µM] (B) in Bohnapfel flesh.

A) B)

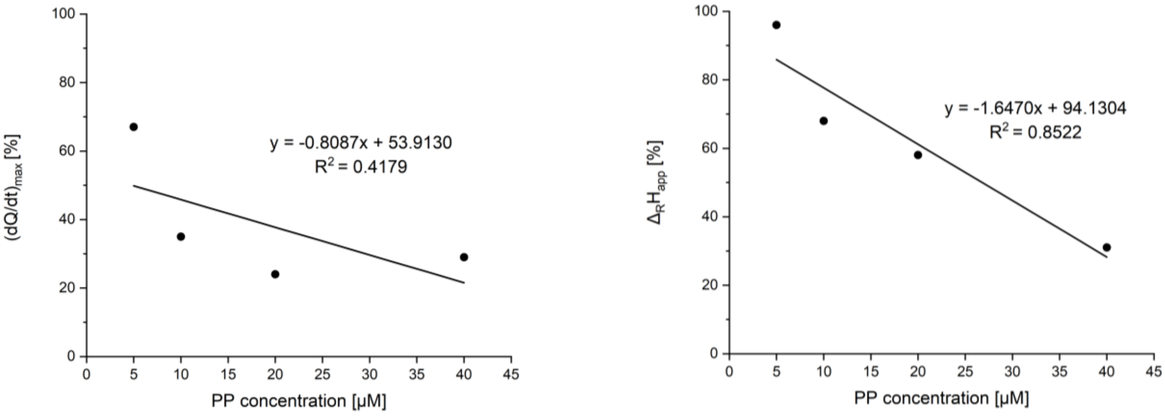

S14: Correlation between the *(dQ/dt)max* [%] and phenolic content [µg/L] in extracts of apple flesh (A) and apple peel (B) from Golden Delicious, Santana, Granny Smith, Gewürzluiken, and Bohnapfel.

A) B)

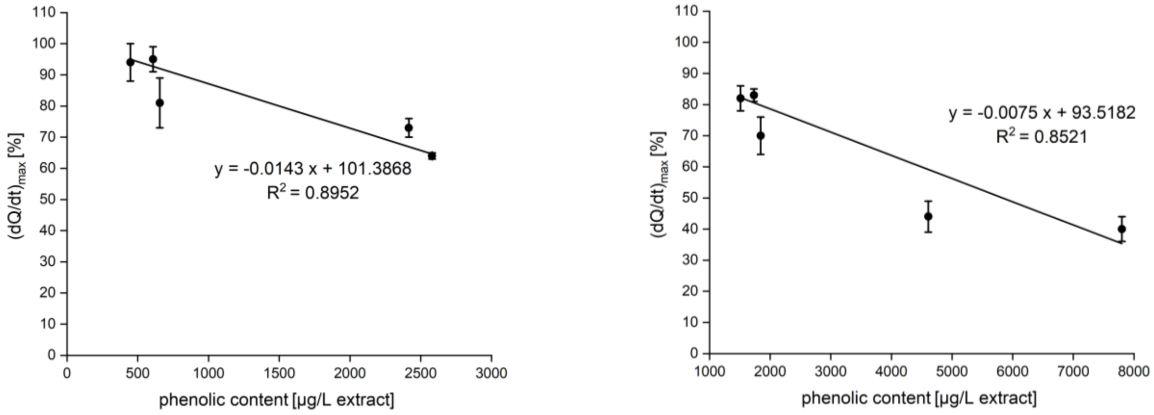

S15: Summary of K_{ic}, K_{iu}, α, IC_{50} values calculated based on the mass concentration for the investigated phenolic structures.

inhibitor	molecular weight [g/mol]	K_{ic} [g/L]	K_{iu} [g/L]	α (K_{ic}/K_{iu})	IC_{50} [g/L]
**control**					
**FA**	194.19	0.24 ± 0.05	0.46 ± 0.29	0.67 ± 0.30	0.35 ± 0.15
**rutin**	610.52	0.22 ± 0.03	0.95 ± 0.53	0.28 ± 0.12	0.47 ± 0.14
**PHL**	436.40	0.18 ± 0.06	0.25 ± 0.06	0.76 ± 0.08	0.22 ± 0.06
**EC**	290.27	0.11 ± 0.05	0.15 ± 0.06	0.74 ± 0.10	0.14 ± 0.06
**CAT**	290.27	0.10 ±0.06	0.14 ± 0.06	0.75 ± 0.40	0.12 ± 0.05
**CA**	354.31	0.07 ± 0.02	0.10 ± 0.04	0.70 ± 0.03	0.07 ± 0.00
**CYD-3-glc**	484.84	0.07 ± 0.01	0.10 ± 0.02	0.71 ± 0.05	0.04 ± 0.01
**Q-3-glc**	464.38	0.05 ± 0.00	0.08 ± 0.01	0.65 ± 0.07	0.07 ± 0.00
**PC B1**	578.52	0.09 ± 0.05	0.13 ± 0.05	0.71 ± 0.20	0.11 ± 0.05
**PC B2**	578.52	0.07 ± 0.02	0.07 ± 0.02	0.94 ± 0.13	0.07 ± 0.02
**EC_{ox}***	290.27	0.02 ± 0.01	0.03 ± 0.00	0.70 ± 0.18	0.03 ± 0.00
**PC C1**	866.77	0.08 ± 0.02	0.09 ± 0.01	0.90 ± 0.12	0.09 ± 0.02
**EGCG**	458.36	0.01 ± 0.00	0.01 ± 0.00	0.73 ± 0.01	0.01 ± 0.00

Data are presented as mean values ± SD (n=4); *data calculated using the molecular weight of monomers.

Bibliography

Julia A. H. Kaeswurm, Lisa Könighofer, Melanie Hogg, Andreas Scharinger and Maria Buchweitz, 2020. Impact of B-Ring Substitution and Acylation with Hydroxy Cinnamic Acids on the Inhibition of Porcine -Amylase by Anthocyanin-3-Glycosides 9, 367.

Kaeswurm, J.A.H., Burandt, M.R., Mayer, P.S., Straub, L.V., Buchweitz, M., 2022. Bioaccessibility of Apple Polyphenols from Peel and Flesh during Oral Digestion. Journal of agricultural and food chemistry 70 (14), 4407–4417. https://doi.org/10.1021/acs.jafc.1c08130.

Kaeswurm, J.A.H., Sempio, R., Manca, F., Burandt, M.R., Buchweitz, M., 2023. Analyzing Bioaccessibility of Polyphenols in Six Commercial and Six Traditional Apples (Malus domestica Borkh.) during In Vitro and Ex Vivo Oral Digestion. Molecular nutrition & food research 67 (22), e2300055. https://doi.org/10.1002/mnfr.202300055.

Rodríguez-Werner, M., Winterhalter, P., Esatbeyoglu, T., 2019. Phenolic Composition, Radical Scavenging Activity and an Approach for Authentication of Aronia melanocarpa Berries, Juice, and Pomace. Journal of food science 84 (7), 1791–1798. https://doi.org/10.1111/1750-3841.14660.
